# Faecal Microbiota Analysis of Piglets During Lactation

**DOI:** 10.3390/ani10050762

**Published:** 2020-04-27

**Authors:** Tanya L. Nowland, Valeria A. Torok, Wai Y. Low, Mary D. Barton, Kate J. Plush, Roy N. Kirkwood

**Affiliations:** 1 School of Animal and Veterinary Sciences, The University of Adelaide, Roseworthy, SA 5400, Australia; roy.kirkwood@adelaide.edu.au; 2 South Australian Research and Development Institute, Food Sciences, SA 5064, Australia; valeria.torok@sa.gov.au; 3 The Davies Research Centre, School of Animal and Veterinary Sciences, University of Adelaide, Roseworthy, SA 5371, Australia; wai.low@adelaide.edu.au; 4 School of Pharmacy and Medical Sciences, University of South Australia, Adelaide, SA 5000, Australia; mary.barton@unisa.com.au; 5 SunPork Group, Murarrie, QLD 4172, Australia; kate.plush@sunporkfarms.com.au

**Keywords:** faecal microbiome transplantation, ceftiofur, antibiotic, bacteria, diversity

## Abstract

Antimicrobial use in animals and the potential development of antimicrobial resistance is a global concern. So, non-antimicrobial techniques for animal disease control are needed. This study aimed to determine whether neonatal ceftiofur (CF) treatment affects piglet faecal microbiomes and whether faecal microbiome transplantation (FMT) can correct it. Two focal piglets per sow were assigned to treatments as follows: cffresh (*n* = 6) received CF (3 mg/kg intramuscular) at 7 d and fresh FMT at 13 d; cffrozen (*n* = 7) received CF at 7 d and frozen FMT at 13 d; CF (*n* = 8) received CF at 7 d and no FMT; and no CF (*n* = 5) received no CF or FMT. DNA was extracted from faecal samples collected on days 7, 13, and 18 for 16S rRNA amplicon analysis. All faecal blends used for the FMT consisted of pooled donor pig faeces at 1:2 ratio with saline, delivered orally at 3 mL/kg. Alpha and beta diversity metrics increased with age (*p* < 0.05). However, no effect of antibiotic or FMT treatment was evident in 13 and 18 d old piglets (*p* > 0.05). Although no effect of treatment was observed, information regarding microbial membership during lactation was gained.

## 1. Introduction

The intestinal tract houses a large, diverse, and relatively stable population of bacteria, archaea, fungi, and viruses, together called the enteric microbiome [1]. Different components of the gut microbiome are involved in numerous functions including the production of antimicrobial compounds [2], nutrient metabolism, degradation of xenobiotics including hormones and development of the immune system [3,4], as well as the established property of competitive exclusion of pathogens [5]. The potential benefits of a normal gut microbiome in animal production have only recently been explored, with the focus historically being on pathogens and their control and particularly the use of antibiotics. While antibiotics are efficacious in pathogen removal, they also impact the normal commensal microbiome. Microbes are needed for maintenance of innate mucosal defenses [6] and antibiotics have been shown to reduce host expression of antimicrobial peptides [7]. Further, microbiome perturbations were shown to occur in pigs after a single amoxicillin injection which were still evident after 5 weeks [8].

An example of a successful alternative to antibiotics in treating human disease is faecal microbiome transplantation (FMT) for the treatment of *Clostridium difficile* infections. The use of antibiotics to treat *C. difficile* infections often results in failure as the antibiotics kill the vegetative bacteria but not their spores [9]. At cessation of antibiotic treatment, spores germinate and recurrent *C. difficile* disease develops. To counter this, FMT has been successfully used to re-establish a “good” gut microbiome to competitively exclude *C. difficile*. This procedure requires that faeces from a healthy donor be inoculated into the patient either orally or via an enema. The use of oral FMT for the treatment of food poisoning or severe diarrhoea was first described by Ge Hong in 4^th^ century China [10]. In recent studies, the use of FMT for the treatment of enteric diseases induced durable changes in the patient’s enteric microbiome, with a more than 90% success rate observable within days and was without adverse side effects [10]. Interestingly, Brandt and Aroniadis [10] also described beneficial effects of FMT on non-enteric diseases such as Parkinson’s disease, insulin resistance, multiple sclerosis, and childhood regressive autism.

The present study aimed to provide a proof of concept for the application of FMT to control pre- and post-weaning enteric disease in pigs. Our intent was not to treat piglet diarrhoea but to confirm an ability to (re)-establish an appropriate enteric microbiome, potentially informing the ability to apply later as a technique to treat animals at times of greatest risk of enteric disease, particularly in early lactation and post-weaning phases. We hypothesised that piglets treated with the antibiotic ceftiofur, a critically important antibiotic, would undergo a reduction in the diversity and quantity of beneficial bacteria and that the treatment of these animals with fresh or previously frozen faeces would result in a re-established microbiome that resembles the microbial composition of the faeces transplanted.

## 2. Materials and Methods

This experiment was conducted at the University of Adelaide Roseworthy piggery with the approval of the University of Adelaide’s Animal Ethics Committee (AEC number: S-2017-063).

### 2.1. Experimental Design and Sample Collection

A total of 15 Large White x Landrace sows (parities 1–2: 1.5 ± 0.5) and their litters were included in the experiment. All sows were group housed and had not received any antibiotics during gestation. Sows were moved into the farrowing shed at day 110 of gestation where they received a commercial lactation diet (14.2 MJ DE/kg) twice daily and had *ad libitum* access to water. Prior to farrowing, sows were fed 2.5 kg/d, which was gradually increased to 7–8 kg/d by day 7 after farrowing. Sows were induced to farrow using cloprostenol two days before their estimated due date. Sows farrowed over two days and piglets cross-fostered as necessary to teat capacity approximately 24 h post-partum. Thereafter, two piglets per sow were randomly selected where possible to be focal pigs and were assigned to one of four treatments:

- Injection of ceftiofur (3 mg/kg intramuscular injection (IM)) at 7 d and fresh FMT at 13 d (*n* = 4 litters, *n* = 6 piglets; cffresh)
- Ceftiofur (3 mg/kg IM) at 7 d and frozen FMT at 13 d (*n* = 4 litters, *n* = 7 piglets; cffrozen)
- Ceftiofur (3 mg/kg IM) at 7 d and no FMT (*n* = 4 litters, *n* = 8 piglets; CF)
- No ceftiofur and no FMT (*n* = 3 litters, *n* = 5 piglets; no CF).

When ceftiofur was administered, all piglets in the litter were treated. FMT was administered to randomly selected piglets within the designated treatment groups. Weaning occurred on day 20 or 21 of age. Faecal samples were collected from each focal piglet at 7 d (prior to ceftiofur administration), 13 d (prior to FMT administration), and at 18 d (prior to weaning). Faeces were collected by separating the focal piglet into a clean crate, stimulating the rectum with a sterile swab and collecting the faeces directly into a sterile container in order to limit contamination. Faeces were placed on ice immediately, transported to the laboratory within 4 h, and stored at −80 °C until required for microbial analyses. For frozen FMT, faeces were collected from 8 clinically healthy 13 d-old donor piglets. After collection, faeces were blended at 1-part faeces, 2-parts saline, with glycerol added to 10%, and stored at −80 °C until required for use the following day. Faecal samples for fresh FMT were collected from the same donor pigs and blended 1:2 in saline without glycerol, however, to ensure the same volume was administered, an additional 10% saline was added. Donor piglets had no previous contact with antibiotics or antibiotic-treated animals. All faecal blends were brought to room temperature before being delivered by oral gavage at 3 mL/kg (Brandt and Aroniadis, 2013). Focal piglets were fasted for 3 h before FMT to minimise gastric acidity. A sample was collected from both the fresh and frozen pooled donor faeces for microbial analysis. 

### 2.2. DNA Extraction and 16S rRNA Amplicon Sequencing

DNA was extracted and purified using a MagMAX^TM^ DNA Multi-Sample Ultra Kit Protocol for Faecal Samples (ThermoFisher Scientific, Australia) following the manufacturer’s instructions. 16S rRNA metagenomic sequencing and library preparations were performed at the AMRID Laboratory at Murdoch University on the Illumia MiSeq platform following the “16S Metagenomic Sequencing Library Preparation” guide [11]. The forward primer (5’ TCG TCG GCA GCG TCA GAT GTG TAT AAG AGA CAG CCT ACG GGN GGC WGC AG) and reverse primer (5’ GTC TCG TGG GCT CGG AGA TGT GTA TAA GAG ACA GGA CTA CHV GGG TAT CTA ATC C) were used to amplify the V3 through V4 hypervariable regions of the 16S rRNA gene. The obtained reads are available under the accession number PRJNA622643 of the Sequence Read Archive (SRA) of the NCBI. Bioinformatic analysis of raw sequence data was done by the Australian Genome Research Facility as follows. The paired-end sequences were merged by aligning the forward and reverse reads using PEAR [12] (version 0.9.5) and the primers were identified and trimmed. All trimmed sequences were processed using Quantitative Insights into Microbial Ecology (QIIME 1.8) [13] USEARCH (version 8.0.1623) [14,15] and UPARSE software [16]. Sequences were quality filtered, full length duplicate sequences were removed and sorted by abundance. Singletons or unique reads in the dataset were discarded using USEARCH tools. Additionally, chimeric sequences were clustered and removed using “rdp_gold” database as the reference. Sequences were grouped into operational taxonomic units (OTUs) based on 97% sequence similarity. Using QIIME, taxonomy was assigned using the Silva database (Version 132) [17].

### 2.3. Statistical Analysis

All weight data were analysed using SPSS, version 26 (IBM, Armonk, NY, USA). All data were tested for normality of residuals and outliers before analysis. A linear mixed model was used to assess the effect of treatment on piglet weight and average daily gain. The fixed effects included in the model were sex, age (7, 13 or 18 days), litter size and treatment (CF, no CF, cffresh, cffrozen). Age was fitted as a repeated measure and sow was included as a random effect. Data are expressed as estimated marginal means ± SEM.

The faecal 16S rRNA bacterial taxonomic data were analysed using multivariate statistical techniques (PRIMER6, PRIMER-E Ltd., Ivybridge, UK). Bray–Curtis measures of similarity [18] were calculated to examine similarities between faecal bacterial communities of piglets from the 16S rRNA data matrices, following standardisation and fourth-root transformation. Analysis of similarity (ANOSIM) [19] was used to test if faecal bacterial communities were significantly different between treatment and age. Similarity percentages (SIMPER) [19] analyses were done to determine which individual bacterial taxa contributed most to the overall dissimilarity among age groups. The overall average dissimilarity between faecal bacterial communities of piglets on the treatments were calculated. The percent contributions of significant OTUs (average dissimilarity/standard deviation > 1) to the top 70% of the average dissimilarities were calculated. Unconstrained ordinations were done to graphically illustrate relationships between treatments using non-metric multidimensional scaling (nMDS) [20,21,22] and principal coordinate analysis (PCO) [23]. Subsets of OTUs found to best represent results from ordinations on the full set of OTU data were also determined by using the BVSTEP procedure [24] on a random selection of starting variables. Matches of ordination produced from the subset of OTUs to the full set of OTUs were determined by Spearman rank correlation (Rho) of elements from the two underlying Bray-Curtis similarity matrices.

Alpha diversity metrics were calculated using the Shannon diversity (H’) index, Pielou’s Evenness (J’) and Number of taxa (S) using DIVERSE (PRIMER6 PRIMER-E Ltd., Ivybridge, UK). Using the Shapiro-Wilk test implemented within the RStudio software (Version 1.1.456, Boston, MA, USA), those alpha diversity metrics that were found to be not normally distributed were analysed using a non-parametric analysis, the Kruskal-Wallis test, with corrections for multiple tests using false discovery rate (FDR) with p-value threshold of 0.05. Alpha diversity metrics were found to be normally distributed and were analysed using analysis of variance (ANOVA).

## 3. Results

### 3.1. Body Weight

No significant difference between weights were visible at 13 days of age for animals treated with CF (5.42 ± 0.18 kg) vs those not treated with CF (5.04 ± 0.37 kg; *p* > 0.05). Additionally, no treatment differences existed between treatments at 18 days of age (no CF: 6.82 ± 0.45 kg, CF: 7.22 ± 0.34 kg, cffresh: 7.09 ± 0.39 kg, cffrozen: 6.49 ± 0.36 kg; *p* > 0.05) or for average daily gain to 13 or 18 days of age (*p* > 0.05).

### 3.2. Treatment Effects on Diversity Metrics

No significant differences were observed between genera for ceftiofur (CF) and non-ceftiofur (no CF)-treated animals for beta diversity metrics at 13 days of age (Global R = 0.181, *p* = 0.144). Additionally, no treatment differences existed at 18 days of age for beta diversity metrics (Global R = 0.033, *p* = 0.255). When assessing bacterial genera richness as measured by Shannon’s diversity index, Pielou’s Evenness and the number of genera, no treatment differences were observed between CF and no CF-treated animals at 13 days and no treatment differences were observed at 18 days of age (*p* > 0.05).

### 3.3. Age Effects on Diversity Metrics

**Figure 1:**
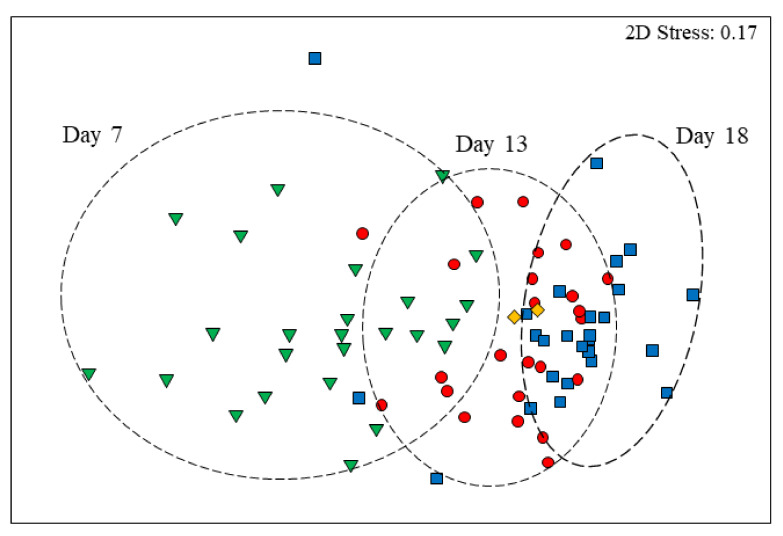
Non-metric multidimensional scaling (nMDS) ordination of faecal bacterial genera from piglets at 7 (inverted triangle), 13 (circle), and 18 (square) days of age along with donor piglets (diamond). All nMDS ordinations attempt to place all samples in an arbitrary two-dimensional space such that their relative distances apart match the corresponding pairwise similarities. Hence, the closer the two samples are in the ordination, the more similar their overall bacterial communities. “Stress” values (Kruskal’s formula 1) reflect the difficulty involved in compressing the sample relationship into the two-dimensional ordination.

**Figure 2:**
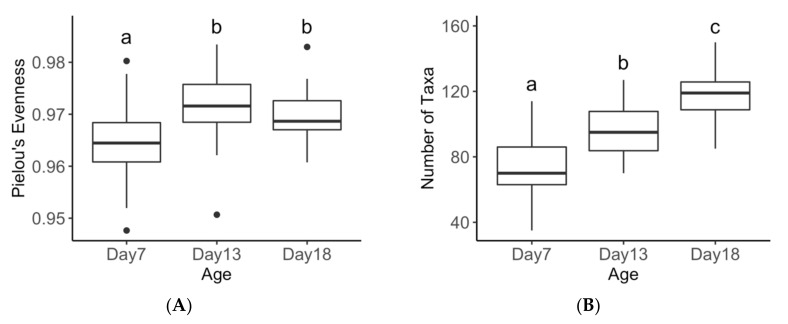

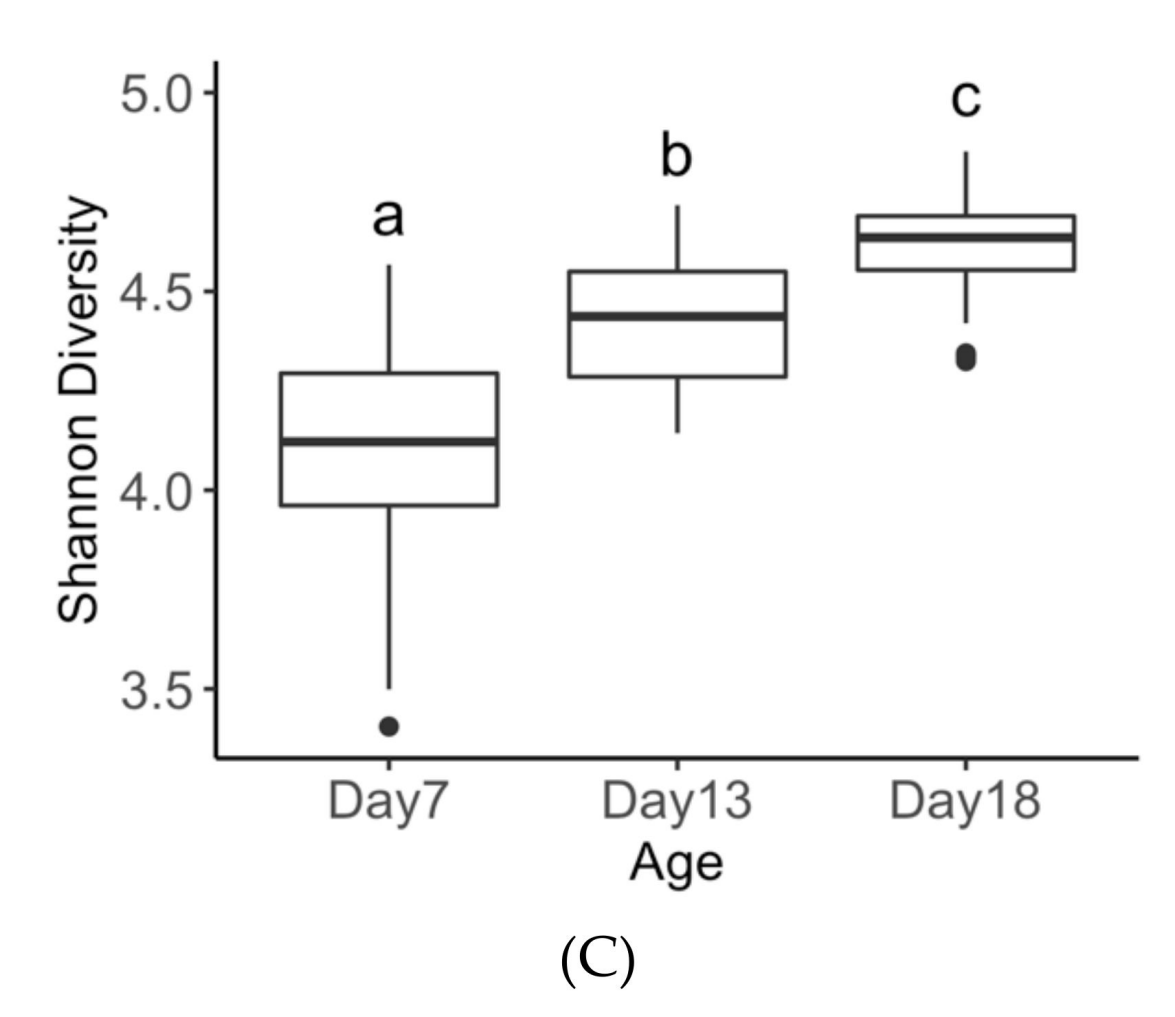
Comparison of bacterial Pielou’s Evenness (**A**), Number of taxa (**B**) and Shannon Diversity (**C**) between piglets at age 7, 13, and 18 days at genus level. Subscripts of differing letters are significantly different from one another (P < 0.05).

Faecal bacterial genera significantly differed with age (Global R = 0.411, *p* = 0.001) with all pairwise comparison being significantly different (day 7 versus day 18 R = 0.635, *p* = 0.001; day 7 versus day 13 R = 0.494, *p* = 0.001; and day 13 versus day 18 R = 0.121 *p* = 0.001). These age-related differences are graphically presented for the bacterial taxa at the genus level in Figure 1, which also shows the donor faeces in relation to all piglets in the study and proximity to 13-day old piglet faecal microbiota. Bacterial genera richness, as measured by Shannon’s diversity index and the number of genera, significantly increased with age (*p* < 0.001; Figure 2). Bacterial community evenness also increased with age with significant differences observed between day 7 and day 13 or day 18 (*p* = 0.002; Figure 2).

### 3.4. Age-Related Taxonomic Composition of Bacterial Communities

**Figure 3:**
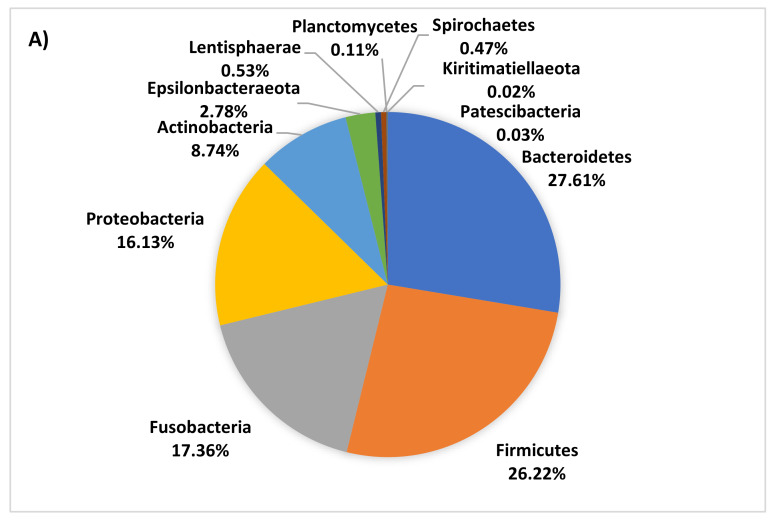

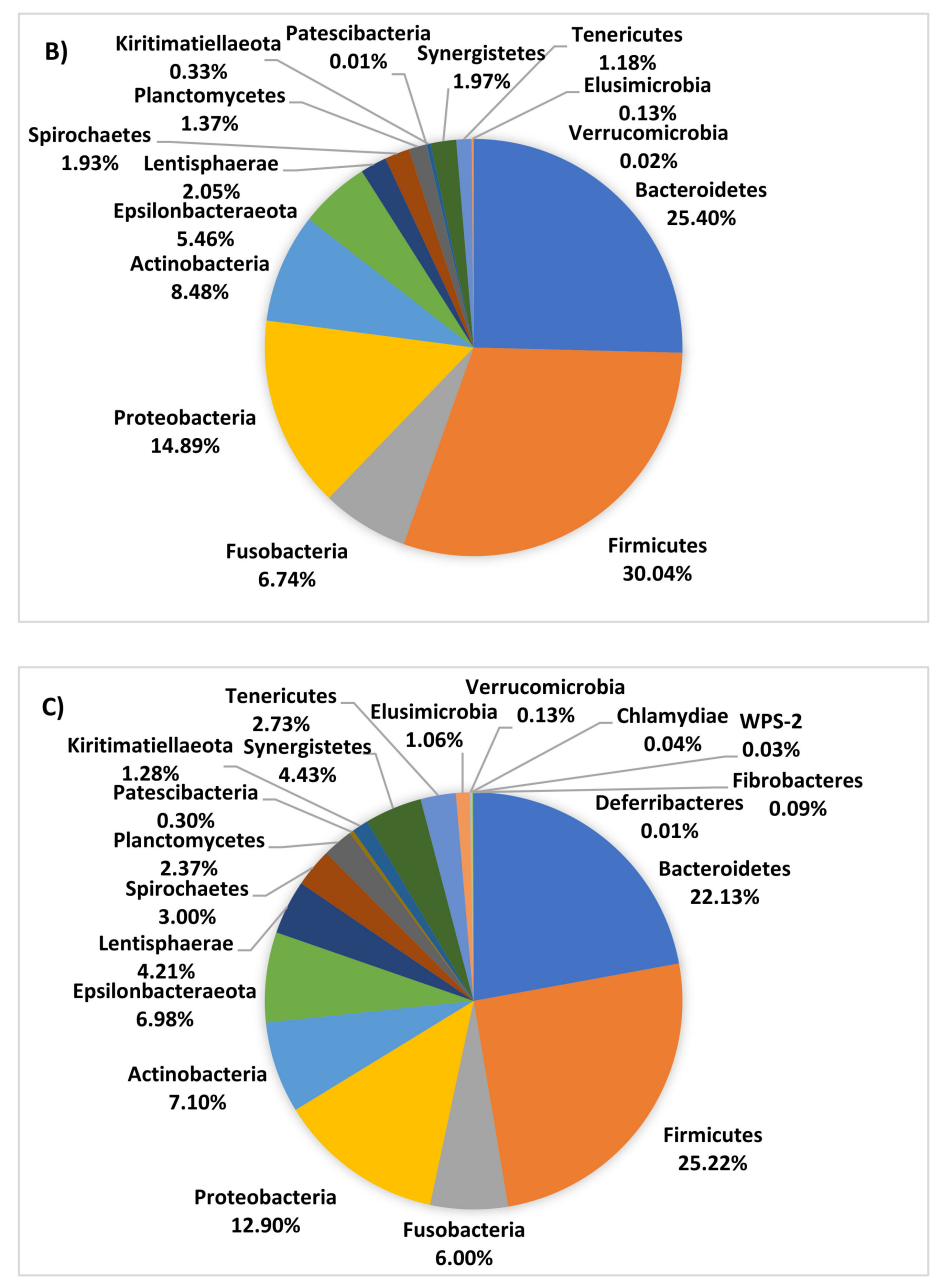
Pie charts of faecal bacteria phyla present at 7 (**A**), 13 (**B**), and 18 (**C**) days of age.

The dominant phyla in piglet faecal microbiota were Bacteroidetes, Firmicutes, Fusobacteria, Proteobacteria, Actinobacteria and Epsilonbacteraeota. However, the proportion of these phyla decreased with age, collectively representing 98%, 91%, and 80% of the microbiota at day 7, 13, and 18, respectively. As piglets aged, both the number and overall proportion of less dominant phyla increased (Figure 3). The average dissimilarity in bacterial phyla between age groups ranged from 23 to 28%. The main phyla driving significant change in faecal microbiota between 7 and 18 days of age were increases in Synergistetes, Epsilonbacteraeota, Lentisphaerae, Spirochaetes, Tenericutes, Firmicutes, and Planctomycetes and decreases in *Fusobacteria* and *Proteobacteria* at 18 days of age.

**Figure 4:**
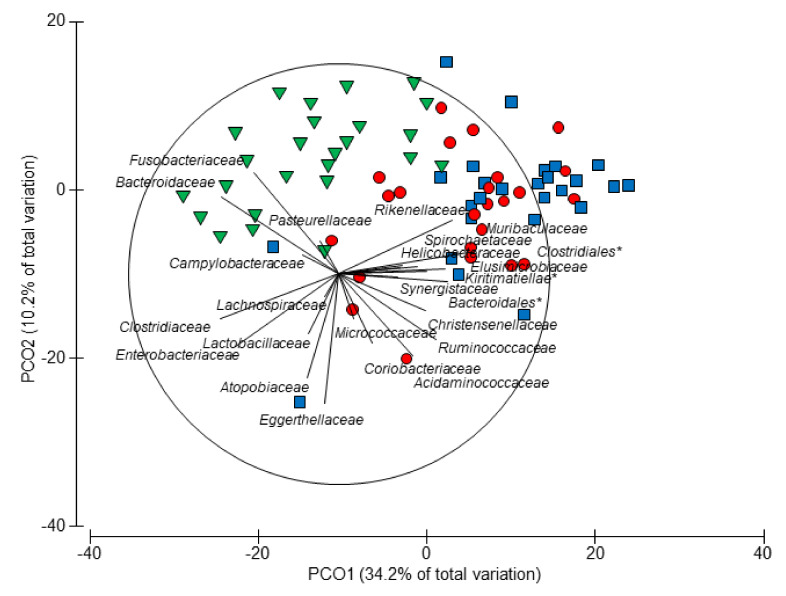
Principal coordinate analysis (PCO) ordination of faecal bacterial families from piglets at 7 (inverted triangle), 13 (circle), and 18 (square) days of age. Overlaid onto the PCO are vectors of the subset of 24 taxa identified by the BVSTEP procedure (Rho = 0.951, P = 0.001) to best represent the overall community pattern from the full set of 81 identified families. Vectors indicate the association of the families with a particular diet. * Uncharacterised family.

The average dissimilarity in bacterial families between piglets aged 7 and 18 days was 36%. Nine age-associated families were confirmed by both the SIMPER (Table 1) and BVSTEP (Figure 4) analyses, with four (*Fusobacteriaceae, Clostridiaceae, Bacteroidaceae,* and *Enterobacteriaceae*) of these having a strong association with the faecal microbiota of 7 day old piglets and five (*Christensenellaceae*, *Muribaculaceae, Rikenellaceae, Synergistaceae* and *Spirochaetaceae*) having a strong association with the faecal microbiota of day 18 piglets. At the genus level, the average dissimilarity in faecal microbiota between piglets aged 7 and 18 days was 51% (Table S1). Of the genera significantly contributing to the top 70% of dissimilarity *Fusobacterium, Bacteriodes*, *Lactobacillus, Escherichia-Shigella, Butyricimonas, Peptostreptococcus, Lachnoclostridium, Actinomyces, Tyzzerella, Veillonella, Ruminococcus, Eisenbergiella, Enterococcus, Streptococcus, Butyricicoccus, Allisonella, Actinobacillus,* and *Hungatella* were more abundant in day 7 piglets and *Prevotella, Campylobacter*, *Pyramidobacter, Alloprevotella, Oscillospira, Roseburia, Alistipes, Dorea, Oscillibacter, Intestinimonas, Treponema, Helicobacter, Sanguibacteroides, Synergistes, Bilophila, Collinsella, Hydrogenoanaerobacterium, Phascolarctobacterium, Vitivallis, Sphaerochaeta, Blautia, Faecalibacterium, Mailhella, Sutterella, Holdemanella, Catenibacterium, Romboutsia,* and *Clostridiodes* were more abundant in the day 18 piglets (Table S1. Of those taxa which could be classified to the species level *Bacteroides fragilis, Bacteroides uniformis, Clostridium perfringens, Bacteroides vulgarus, Escherichia coli, Fusobacterium gastrosuis, Bacteroides plebeius, Actinomyces hyovaginalis, Clostridum baratii, Lactobacillus johnsonii* and *Lactobacillus mucosae* were more abundant in the 7 day old piglets and *Campylobacter jejuni, Megasphaera elsdenii, Lactobacillus reuteri, Lactobacillus salivarius, Lactobacillus coleohominis,* and *Sanguibacteroides justesenii* were more abundant in 18 day old piglets, contributing significantly to the top 50% of dissimilarity between these age groups.

## 4. Discussion

FMT is an effective tool for the treatment of enteric clostridial disease in humans [1,9]. Consequently, this study aimed to provide a proof of concept for the use of FMT in pigs. An antibiotic was administered to piglets with the aim of disrupting their gastrointestinal microbiota and either fresh or frozen FMT was applied to re-establish a normal microbiota. The results showed that faecal diversity increased with age. However, the antibiotic administration had no impact on the faecal microbiota, and in contrast to our hypothesis, FMT had no effect on altering the faecal microbiota of piglets at weaning.

In the present study, the faecal microbiota of piglets increased in diversity and richness as age increased, irrespective of treatment. This finding is supported by the literature [25,26]. It is well established that the period preceding birth is the point whereby human neonates develop their gastrointestinal microbiome [27,28]. As such, an increase in microbial species number and diversity during the weeks following birth is expected. This early period is considered the most critical time for human gastrointestinal microbiome development, with disruptions to the microbiome during this time having consequences for long-term health [29]. Interestingly, we are aware of no research to date that has identified a critical time period for gastrointestinal microbiome development for the pig. However, it is well established that the main factors influencing the development of the microbiota of piglets as they age are the environment and diet to which they are exposed [30,31]. It is evident from the present study that large changes in microbial composition and diversity occur within the first two weeks following birth and then undergoes little change during the last week prior to weaning. It is interesting that such a large shift occurs between 7 and 13 days in the present study as no change in environment occurred during this time and the piglets had no access to creep feed throughout lactation. It is possible that the piglets may have been able to access the sow’s feed, however, previous research investigating creep feed usage suggests that feed consumption is low and variable with it increasing linearly from 2 weeks of age [32]. Although the present study did not go beyond weaning, in other studies investigating the microbiota of piglets post-weaning, the microbiota continues to undergo changes beyond 18 days of age [26,33]. This is to be expected as the diet changes significantly post-weaning. Alternately, piglets are known to exhibit coprophagy [34], which in turn would aid in the development of the microbiota and suggests that this change may be a function of natural gut maturation as piglets age.

One of the main differences observed with age at the phyla level were increased Synergistetes and decreased Fusobacteria. Interestingly, unlike in the present study, Fusobacteria and Synergistetes were not observed in the faeces of 21 day old nursed piglets in a study conducted by Guevarra*,* et al. [26]. Furthermore, Spirochaetes which were classified as one of the main phyla in the study conducted by Guevarra*,* et al. [26], were present only in low amounts in the present study. Additionally, McCormack*,* et al. [35] found similar findings to Guevarra*,* et al. [26], whereby Fusobacteria were only found in small amounts and Synergistetes were not documented in piglets prior to weaning. Although no production differences can be observed between studies, this provides further evidence to demonstrate that the microbiota of individuals not only differs with age but also differs between farms and locations, suggesting environmental and possibly genetic components. Similar to other studies, Bacteriodetes and Firmicutes were the two most abundant phyla present at all age stages prior to weaning. Although the ratio of Bacteriodetes to Firmicutes was similar in the present study, they were present in comparatively much lower amounts.

Of the potentially pathogenic bacteria detected in the faeces of piglets, it is evident that younger aged piglets had significantly more *Escherichia-Shigella* than those at 18 days of age. Other potentially pathogenic bacteria detected were *Streptococcaceae*, *Fusobacterium,* and *Bacteroides* however these are also common gut commensals so their presence would be expected, and it is not until an imbalance of bacteria occurs that they may exhibit a more pathogenic nature. However, *Clostridia* was also detected which has the potential to be an opportunistic pathogen. It is known that an increase in bacterial diversity is generally associated with a reduction in diarrhea in pigs [36] and improved gastrointestinal health in humans [30]. It is important to note that the *E. coli-Shigella* detected may be commensal strains and not necessarily pathogenic, and the fact that these bacteria decrease as pigs age may be a byproduct of the natural increase in microbial diversity as pigs age and their gastrointestinal tract matures.

It is well established that the administration of antibiotics to animals alters their gastrointestinal microbiota, with marked reductions in population diversity being detected in the faeces from 7 days post-treatment [37]. However, this response did not occur within the present study. Those animals that received antibiotics via an intramuscular injection at 7 days of age showed no differences in Shannon diversity, Pielou’s evenness or number of taxa 6 or 11 days after antibiotic administration. This indicates that those animals that were treated with antibiotics had a similar community structure to one another. However, regardless of antibiotic exposure they were both equally as diverse, even in distribution, and had a similar number of taxa composing them. These findings contrast with Gao*,* et al. [37], who observed a significant reduction in Shannon diversity and evenness within the faecal microbiota of pigs provided with in-feed antibiotics 7 days after the beginning of the treatment. Although that study differed from the present study as to the antibiotic type, route, and duration of administration, a change to diversity and evenness of the faecal microbiota would still have been expected. Janczyk*,* et al. [8], noted differences in the faecal microbiota 5 weeks after administering one dose of intramuscular amoxicillin to a 1-day old piglet. However, the latter study did not investigate how quickly the differences became established. More recently, Ruczizka*,* et al. [38] observed differences in the microbiota of piglets given a single intramuscular injection of ceftiofur 12 h post-partum, with differences evident at 12, 28 and 97 days of age. This approach was conducted in a more similar manner to the one implemented in the current study, therefore, it is likely that our inability to detect an effect of antibiotic may be due to a need for a longer period of time after antibiotic administration in order to see a change in the faeces. Additionally, Ruczizka*,* et al. [38], also observed sex-specific differences and had 16 piglets of each sex per treatment. So, the lower number of replicates in the present study may have impacted the results.

FMT has been demonstrated to be an effective tool in the treatment of *Clostridium difficile* infections in humans [9], with studies showing that FMT changes the microbiota of the sick recipient to become more similar to that of the faeces they were treated with [39]. Even more recently within the pig industry, investigation into the use of FMT as a tool for improving feed conversion efficiency has been conducted. However, contradictory results have been observed, with some showing positive and other negative impacts on feed conversion efficiency of the treated pigs. It has been suggested that the donor faeces used impacted the outcomes observed [35,40]. In contrast to previous studies, no differences in faecal microbiota were observed between treatments in the current study. This may be due to not enough time being allowed after FMT for a greater change to be observed, and suggests that the administration of a single FMT to 13-day old piglets does not produce changes in the faecal microbiota to weaning at 18 days of age, or our replicates were insufficient to assess this. Studies conducted in humans investigating FMT as a treatment for *Clostridium difficile* infections found that one to two FMT doses was sufficient [1,9]. However, unlike the studies in humans where the patient’s microbiome would have been unstable due to the excessive antibiotic use beforehand, the microbiota of our recipient piglets would have been relatively stable, and the antibiotic had no impact on the piglet’s faecal microbiota. Therefore, it is likely that the ability for the donor faeces to competitively exclude the bacteria already present within the GIT would have been limited. Furthermore, the donor animals used for the preparation of the FMT were a similar age to those animals that were receiving the FMT. Therefore, because the antibiotics had no effect on the microbiota as expected, the FMT given may not have been different enough from the piglets receiving it to see an effect. Overall, further research is likely necessary in order to understand the best timing for dosing with FMT and the likely number of FMT doses required in order to create a long-lasting change within the microbiota of piglets.

## 5. Conclusions

Significant differences in faecal bacterial communities associated with age were observed. Specifically, a reduction in the dominant phyla, particularly Fusobacteria and Proteobacteria, and an increase in less dominant phyla by 18 days of age. However, the finding that animals treated with the antibiotic ceftiofur had no reduction in alpha diversity metrics was unexpected, as other studies have shown that antibiotics reduced both alpha and beta diversity metrics. Additionally, FMT had no influence on piglets to weaning, which may be attributed to the fact that antibiotic administration did not disrupt the microbiota as initially intended and the donor faeces used may not have been different enough to elicit an effect that was detectable. Although no treatment effect was observed, information regarding microbial membership in the pre-weaning period for piglets was gained.

## Figures and Tables

**Table 1:** Family contributing to the top 70% of significant dissimilarity of bacteria between 7 and 18 day old piglets as determined by SIMPER. Overall, average dissimilarity between ages is 36%.

	Day 7	Day 18	
Family	Average Abundance	Average Abundance	%
Uncharacterised Clostridiales	0.22	1.21	4.27
*Christensenellaceae*	0.38	1.37	8.47
*Fusobacteriaceae*	1.71	0.88	12.59
*Muribaculaceae*	0.86	1.52	16.25
*Clostridiaceae*	1.35	0.61	19.56
*Prevotellaceae*	1.32	1.45	22.5
Uncharacterised Bacteroidales	0.06	0.7	25.32
*Synergistaceae*	0.02	0.69	28.11
*Bacteroidaceae*	2.1	1.63	30.86
*Lactobacillaceae*	1.4	1.24	33.52
*Campylobacteraceae*	0.58	0.83	36.13
*Enterobacteriaceae*	1.37	1.09	38.62
*Rikenellaceae*	0.88	1.24	41.03
*Marinifilaceae*	0.83	0.9	43.36
*Ruminococcaceae*	1.61	1.94	45.44
*Actinomycetaceae*	0.58	0.28	47.37
*Spirochaetaceae*	0.17	0.53	49.29
*Victivallaceae*	0.16	0.5	51
*Enterococcaceae*	0.36	0.31	52.7
*Helicobacteraceae*	0.12	0.41	54.31
*Veillonellaceae*	0.88	0.68	55.87
*Lachnospiraceae*	1.7	1.75	58.98
*Oligosphaeraceae*	0.04	0.38	60.52
*Coriobacteriaceae*	0.51	0.61	62.05
*Acidaminococcaceae*	0.86	1.13	63.55
Uncharacterised Mollicutes	0	0.36	65.05
*Pirellulaceae*	0.09	0.38	66.52
*Streptococcaceae*	0.82	0.58	67.94
Uncharacterised Bradymonadales	0.12	0.33	69.34
*Pasteurellaceae*	0.63	0.53	70.73

## Supplementary Materials

The following are available online at https://www.mdpi.com/2076-2615/10/5/762/s1, Table S1: Genus contributing to the top 70% of significant dissimilarity of bacteria between day 7 and 18 age groups as determined by SIMPER. Overall average dissimilarity between ages is 51%.
