# Supplementary material for: Faecal Microbiota Analysis of Piglets During Lactation
Source: Animals (Basel). 2020 Apr 27;10(5):762. doi: 10.3390/ani10050762 (PMC7277143; doi:10.3390/ani10050762)
Supplement: Supplementary file 1 [file animals-10-00762-s001.pdf]

Table S1: Genus contributing to the top 70% of significant dissimilarity of bacteria between day 7 and 18 age groups as determined by SIMPER. Overall average dissimilarity between ages is 51%.

Phyla	Class	Order	Family	Genus	Day 7 Average Abundance	Day 18 Average Abundance	%
Actinobacteria	Actinobacteria	Actinomycetales	*Actinomycetaceae*	*Actinomyces*	0.57	0.24	0.74
	Coriobacteriia	Coriobacteriales	*Coriobacteriaceae*	*Collinsella*	0.51	0.61	0.57
Bacteroidetes	Bacteroidia	Bacteroidales	*Bacteroidaceae*	*Bacteroides*	2.1	1.63	1.03
			Marinifilaceae	*Butyricimonas*	0.82	0.81	0.9
				Sanguibacteroides	0.08	0.42	0.59
				CAG-873	0.01	1.1	1.68
			Marinifilaceae		0.38	0.78	0.91
			Marinifilaceae		0.78	1.04	0.97
			p-2534-18B5 gut group		0.06	0.7	1.04
			Prevotellaceae	*Alloprevotella*	0.46	0.83	0.89
				Prevotella 2	0.95	1.11	1.3
				Prevotella 7	0.22	0.42	0.56
				Prevotellaceae NK3B31 group	0.62	0.68	0.77
				Prevotellaceae UCG-003	0.26	0.64	0.89
				Prevotellaceae UCG-004	0.11	0.48	0.68
			Prevotellaceae		0.64	0.52	0.89
			Prevotellaceae		0.27	0.42	0.55
			Rikenellaceae	*Alistipes*	0.39	0.62	0.77
				dgA-11 gut group	0.04	0.38	0.56
				RC9 gut group	0.79	1.17	0.95
Epsilonbacteraeota	Campylobacteria	Campylobacterales	*Campylobacteraceae*	*Campylobacter*	0.58	0.83	0.97
			Helicobacteraceae	*Helicobacter*	0.12	0.41	0.6
Firmicutes	Bacilli	Lactobacillales	*Enterococcaceae*	*Enterococcus*	0.36	0.31	0.64
			Lactobacillaceae	*Lactobacillus*	1.4	1.24	1
			Streptococcaceae	*Streptococcus*	0.82	0.58	0.53
Firmicutes	Clostridia	Clostridiales	*Christensenellaceae*	*Christensenellaceae R-7 group*	0.38	1.36	1.56
			Clostridiaceae	*Clostridium sensu stricto 1*	1.16	0.58	1

	Clostridium sensu stricto 2	0.92	0.14	1.31
Clostridiales vadinBB60 group		0.05	0.67	0.97
Clostridiales vadinBB60 group		0.18	1.15	1.56
Family XIII	*[Eubacterium] nodatum group*	0.29	0.46	0.51
	Family XIII AD3011 group	0.15	0.59	0.72
Lachnospiraceae	*[Eubacterium] fissicatena group*	0.92	0.61	0.67
	[Ruminococcus] gauvreauii group	0.32	0.53	0.68
	Blautia	0.44	0.49	0.51
	Dorea	0.51	0.72	0.72
	Eisenbergiella	0.73	0.45	0.65
	Hungatella	0.56	0.43	0.48
	Lachnoclostridium	1.44	1.43	0.75
	Lachnospiraceae FCS020 group	0.15	0.39	0.53
	Lachnospiraceae UCG-002	0.07	0.31	0.47
	Lachnospiraceae UCG-010	0.11	0.4	0.56
Lachnospiraceae		0.2	0.74	0.98
	Roseburia	0.47	0.51	0.78
	Tyzzerella	0.59	0.31	0.72
Peptostreptococcaceae	*Clostridioides*	0.3	0.13	0.47
	Peptostreptococcus	0.66	0.21	0.81
	Romboutsia	0.48	0.55	0.47
Ruminococcaceae	*[Eubacterium] coprostanoligenes group*	1.14	1.14	0.62
	Butyricicoccus	0.59	0.48	0.51
	Faecalibacterium	0.08	0.33	0.5
	GCA-900066225	0.23	0.35	0.45
	Hydrogenoanaerobacterium	0.38	0.64	0.56
	Intestinimonas	0.62	0.97	0.66
	Oscillibacter	0.12	0.52	0.7
	Oscillospira	0.22	0.65	0.83
	Ruminiclostridium 9	0.57	0.83	0.71
	Ruminococcaceae NK4A214 group	0.51	1.01	0.9
	Ruminococcaceae UCG-002	0.75	1.24	1.04
	Ruminococcaceae UCG-003	0.26	0.54	0.72
	Ruminococcaceae UCG-005	0.22	0.83	0.98

				Ruminococcaceae UCG-010	0.46	0.83	0.74
				Ruminococcus 2	0.92	0.81	0.68
				Subdoligranulum	0.08	0.45	0.66
				UBA1819	0.6	0.48	0.45
			Ruminococcaceae		0.77	0.97	0.68
	Erysipelotrichia	Erysipelotrichales	*Erysipelotrichaceae*	*Catenibacterium*	0.17	0.33	0.48
				Erysipelotrichaceae UCG-004	0.19	0.47	0.65
				Holdemanella	0.52	0.56	0.49
			Erysipelotrichaceae		0.12	0.71	0.97
	Negativicutes	Selenomonadales	*Acidaminococcaceae*	*Phascolarctobacterium*	0.85	1.1	0.54
			Veillonellaceae	*Allisonella*	0.31	0.12	0.49
				Megasphaera	0.18	0.43	0.68
				Veillonella	0.77	0.42	0.69
Fusobacteria	Fusobacteriia	Fusobacteriales	*Fusobacteriaceae*	*Fusobacterium*	1.71	0.88	1.54
Lentisphaerae	Lentisphaeria	Victivallales	*Victivallaceae*	*Victivallis*	0.12	0.38	0.54
	Oligosphaeria	Oligosphaerales	*Oligosphaeraceae*	*Z20*	0.04	0.38	0.57
Planctomycetes	Planctomycetacia	Pirellulales	*Pirellulaceae*	*p-1088-a5 gut group*	0.09	0.38	0.55
Proteobacteria	Deltaproteobacteria	Bradymonadales			0.12	0.33	0.52
		Desulfovibrionales	*Desulfovibrionaceae*	*Bilophila*	0.32	0.59	0.57
				Mailhella	0.17	0.36	0.5
			Desulfovibrionaceae		0.06	0.31	0.47
	Gammaproteobacteria	Betaproteobacteriales	*Burkholderiaceae*	*Sutterella*	0.8	0.73	0.49
		Enterobacteriales	*Enterobacteriaceae*	*Escherichia-Shigella*	1.37	1.09	0.94
		Pasteurellales	*Pasteurellaceae*	*Actinobacillus*	0.58	0.5	0.48
Spirochaetes	Spirochaetia	Spirochaetales	*Spirochaetaceae*	*Sphaerochaeta*	0.13	0.36	0.53
				Treponema 2	0.08	0.45	0.64
Synergistetes	Synergistia	Synergistales	*Synergistaceae*	*Pyramidobacter*	0.02	0.6	0.91
				Synergistes	0	0.37	0.57
Tenericutes	Mollicutes	Mollicutes RF39			0	0.36	0.55
